# Deciphering Role of Wnt Signalling in Cardiac Mesoderm and Cardiomyocyte Differentiation from Human iPSCs: Four-dimensional control of Wnt pathway for hiPSC-CMs differentiation

**DOI:** 10.1038/s41598-019-55620-x

**Published:** 2019-12-18

**Authors:** Meng Zhao, Yawen Tang, Yang Zhou, Jianyi Zhang

**Affiliations:** Department of Biomedical Engineering, School of Medicine, School of Engineering, University of Alabama at Birmingham, Birmingham, AL35233 USA

**Keywords:** Stem-cell differentiation, Heart failure, Growth factor signalling

## Abstract

Differentiation of cardiomyocytes (CMs) from human induced pluripotent stem cells (hiPSCs) is critically dependent upon the regulation of the Wnt signaling pathway. The mechanisms remain unclear with regard to the dose and timing of each differentiation inducer, and the interaction of the inducers that regulate the Wnt in mesendoderm specification to cardiac mesoderm. Consequently, it remains far from optimal in differentiation efficiency and consistency from hiPSC lines to CMs. Here, we have carefully deciphered the role of Wnt signaling pathway manipulation on mesoderm specification in a dosage and time dependent manner. To examine the hypothesis of that fate specification of hiPSC-CMs differentiation is dictated by temporal and spatial factors that regulate Wnt, we evaluate hiPSC-CM differentiation with: (1) two-phase modulation of Wnt, (2) dosage variant of GSK3β inhibitors, (3) treatment with insulin, and (4) 3-dimentional suspension culture environment on iPSC-CM differentiation. The results highlight the importance of mesendoderm specification to cardiac mesoderm, which needs precisely regulation of Wnt in a dosage dependent and temporal on/off manner. This temporal regulation dictates the final efficiency and purity of derived cardiomyocytes. After the initial activation of Wnt signaling pathway to generate mesendoderm, the maintenance of Wnt signaling at an appropriate dose is critical to direct the cell fate into cardiac mesoderm. Otherwise, lower Wnt signals lead to definitive endoderm and higher Wnt signals induce presomitic mesoderm differentiation. The precisely specification of cardiac mesoderm results in not only greater than 90% of cTnT^+^ cardiomyocytes but also high cardiomyocytes yield under both monolayer and suspension culture conditions. Thus, the current findings provide critical insights to decipher the temporal mechanism of Wnt activation in regulation of hiPSC-CMs differentiation, and more importantly provide the guidelines for the consistent and high-yield and high-quality hiPSC-CMs production in cardiovascular research.

## Introduction

Mammalian cardiomyocytes (CMs) exit the cell cycle shortly after birth; thus, contractile tissue that is damaged by an infarct event or myocardial disease cannot be replaced via the proliferation of surviving CMs. This limited regenerative capacity has driven researchers to develop new sources of CMs for treating patients with myocardial disease, as well as disease modeling and drug discovery^1^. Human induced-pluripotent stem cells (hiPSCs)^2^ are among the most promising sources, because their proliferative capacity is essentially unlimited, and they can be differentiated into any type of somatic cells. Protocols for differentiating hiPSCs into CMs are becoming increasingly sophisticated^3^, but the efficiency and reproducibility of conventional protocols can be substantially influenced by genetic or epigenetic factors associated with the tissues from which the hiPSCs were generated^4–6^, and whether these protocols can be adapted to produce the large number of cells needed to meet the anticipated clinical demand has yet to be determined.

Although mechanisms remain not clearly defined in terms of timing and doses of each differentiation inducers, one of the most effective hiPSC-CM differentiation protocols is designed to recapitulate the biphasic role of Wnt/β-catenin signaling during embryonic cardiogenesis and prenatal growth^7^. At embryonic day 5.75 in developing mice, Wnt induces the expression of mesendodermal markers such as Brachyury (Bry) and Eomesodermin (EOMES), which directly activate the primary cardiac mesoderm regulator mesoderm posterior BHLH transcription factor 1 (Mesp1)^8^; then, Mesp1 activates Dickkopf Wnt Signaling Pathway Inhibitor 1 (DKK1), which subsequently inhibits Wnt and drives cardiac-lineage specification^9^. In differentiating hiPSC-CMs, Wnt activation is achieved indirectly via the inhibition of glycogen synthase kinase 3β (Gsk3β) (i.e., the Gi step), whereas Wnt inhibition (Wi) is induced directly with a Wnt inhibitor. The GiWi protocol is simpler and less costly than methods that rely on transcription factors and other proteins, because both Wnt activation and inhibition are induced with pharmacological products, and has been used to induce hiPSC-CM differentiation in both 2-dimensional (i.e., monolayers)^3^ and 3-dimensional (i.e., embryoid bodies/spheroids)^10,11^ culture systems. However, the efficiency of the conventional GiWi protocol appears to be greater for hiPSCs derived from cardiac (hciPSCs), rather than dermal (hdiPSCs) fibroblasts^12^ and may also vary depending on how many times the hiPSCs were passaged before differentiation was induced^4–6^.

In this study, we hypothesized that fate specification of hiPSC-CMs differentiation is dictated by temporal and spatial factors Wnt activation. We conducted a thorough investigation of the role of Wnt pathway activity during the Gi phase of the GiWi protocol. Our initial results suggested that limiting Wnt activation to just the first 24 hours of the 72-hour Gi period failed to sufficiently induce mesodermal specification before the Wnt inhibition phase was initiated, so we developed a new method, the Gi(I/M)Wi protocol, in which Wnt activation was initiated (I) with a high dose of the Gsk3β inhibitor CHIR99021 (CHIR) for 24 hours and then maintained (M) with a lower CHIR dose until the Wnt inhibitor IWR1 was added 48 hours later. Furthermore, we investigated additional adaptable method (i.e. suspension culture) and parameter (i.e. insulin treatment) for the new Gi(I/M)Wi protocol. Thus, these results demonstrate a critical temporal mechanism of Wnt activation in fate speciation of hiPSC-CM differentiation. The flexibility of this new protocol established here, enables the magnitude of Wnt activity to be adjusted to optimize hiPSC-CM differentiation across multiple hiPSC lines and culture conditions.

## Results

### The Gi(I/M)Wi protocol improves hiPSC-CM differentiation by using a low CHIR maintenance dose to preserve Wnt activation throughout the Gi phase

**Figure 1:**
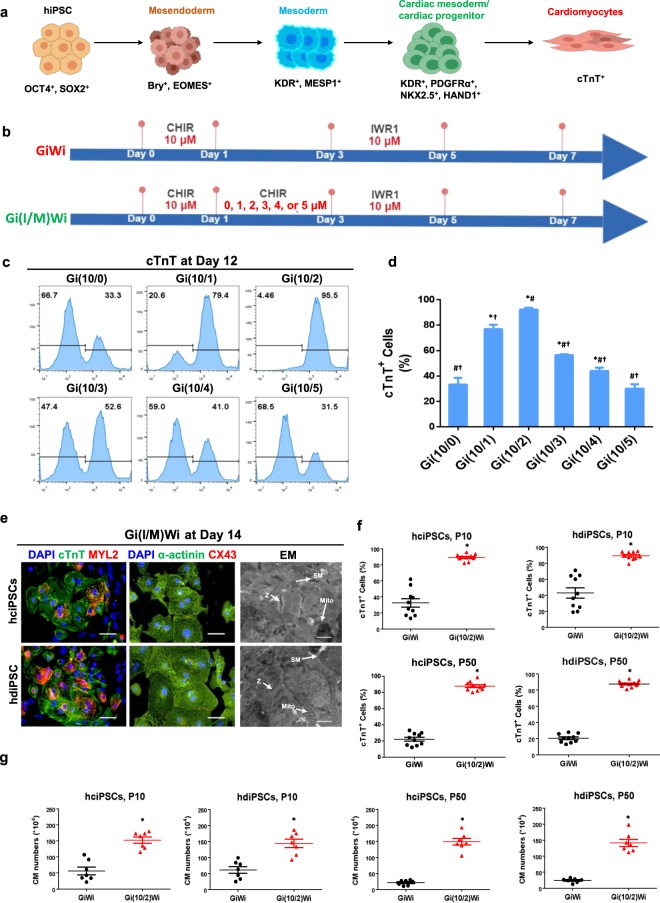
The Gi(I/M)Wi hiPSC-CM differentiation protocol is highly efficient for hiPSCs of different origins and passages. **(a)** The differentiation of hiPSCs into CMs progresses through stages of mesendoderm, mesoderm, and cardiac mesoderm/CPC specification, and each stage is associated with the expression of specific sets of genetic markers. **(b)** The GiWi and Gi(I/M)Wi protocols are illustrated schematically, including the time points and concentrations of CHIR and IWR1 treatment. In the Gi(I/M)Wi protocol, the cells were differentiated with the following initial (I) and maintenance (M) CHIR doses: Gi(10/0), I = 10 μM and M = 0 μM; Gi(10/1), I = 10 μM and M = 1 μM; Gi(10/2), I = 10 μM and M = 2 μM; Gi(10/3), I = 10 μM and M = 3 μM; Gi(10/4), I = 10 μM and M = 4 μM; Gi(10/5), I = 10 μM and M = 5 μM. **(c)** Twelve days after differentiation was initiated, expression of the CM marker cTnT was evaluated in cells from all six Gi(I/M)Wi treatment groups via flow cytometry; then, **(d)** the proportion of cells that expressed cTnT was calculated and presented as a percentage of the total number of cells (n = 3 different batches of differentiated cells). *P < 0.05 vs. Gi(10/0); ^#^P < 0.05 vs. Gi(10/1); ^†^P < 0.05 vs. Gi(10/2).**(e)** hiPSCs reprogrammed from cardiac (hciPSCs) or dermal (hdiPSCs) fibroblasts were differentiated via the Gi(I/M)Wi protocol with CHIR initiation and maintenance doses of 10 μM and 2 μM respectively. Fourteen days after differentiation was initiated, the cells were immunofluorescently stained and imaged for the expression of cTnT, MYL2, α-actinin, and Cx43 (bars = 50 μm) and also verified by transmission electron microscopy (SM: sarcomere, Z: Z-band, Mito: mitochondrion; bars = 2 μm). **(f,g)** hciPSCs and hdiPSCs were passaged 10 (P10) or 50 (P50) times and then differentiated via the GiWi or Gi(10/2**)**Wi protocols; then, cTnT expression was evaluated via flow cytometry. **(f)** The proportion of cells that expressed cTnT was presented as a percentage of the total number of cells (n = 10 different batches of differentiated cells), and **(g)** the total number of cTnT-expressing cells per well was quantified (n = 7 different batches of differentiated cells). *P < 0.05 vs GiWi.

The differentiation of hiPSCs into CMs progresses through multiple steps of cell-fate determination, and each stage is accompanied by the expression of identity-specific marker genes. hiPSCs express the pluripotency genes octamer-binding transcription factor 4 (OCT4) and SRY-box 2 (SOX2), and cells in subsequent stages of differentiation can be identified by the expression of Bry and EOMES for mesendodermal cells; MESP1 and kinase insert domain receptor (KDR) for mesodermal cells; KDR, platelet derived growth factor receptor α (PDGFRα), NK2 homeobox 5 (NKX2.5), and heart and neural crest derivative 1 (HAND1) for cells of the cardiac mesoderm and cardiac progenitor-cell lineages; and, finally, cardiac troponin T (cTnT) for cardiomyocytes (Fig. 1a). One of the most well-established and widely used protocols for generating hiPSC-CMs uses two small molecules, the GSK3β inhibitor (Gi) CHIR99021 (CHIR) and the Wnt inhibitor (Wi) IWR1 (i.e., the GiWi protocol). CHIR treatment activates Wnt signaling and continues for 24 hours to induce mesendodermal/mesodermal specification; then, the cells are maintained in the absence of CHIR for 48 hours before IWR1 is added to inhibit Wnt signaling and promote the cardiac/cardiomyocyte phenotype (Fig. 1b). However, reproducibility of the traditional GiWi protocol is poor and often yields heterogeneous results across different cell lines and under varying culture conditions^13,14^. Thus, we began our investigation of the role of Wnt signaling in hiPSC-CM differentiation by evaluating the expression of mesendodermal and mesodermal markers 1–3 days after differentiation was initiated, but before the Wnt inhibitor was added at Hour 72.

hiPSCs that had been reprogrammed from cardiac fibroblasts (hciPSCs) were treated with 10 μM CHIR for 24 hours, which activated β-catenin (a component of the Wnt-signaling pathway); then, β-catenin re-localized into the nucleus (Supplemental Fig. 1a), and the cells rapidly differentiated into the mesendodermal lineage, as evidenced by declines in the expression of OCT4 and SOX2 (Supplemental Fig. 1b) and dramatic increases in the proportion of cells that expressed EOMES (Supplemental Fig. 1c) and Bry. However, MESP1 expression was not detectable until Day 3 of differentiation (Supplemental Fig. 1d) and even then, MESP1 levels remained low, which suggested that for maximum mesodermal and cardiac mesodermal specification, Wnt signaling needed to be maintained and optimized in the differentiating cells before treatment with the Wnt inhibitor IWR1. Thus, we investigated whether the efficiency of hiPSC-CM differentiation could be improved by maintaining the cells in low concentrations of CHIR after the high-dose initiation period (i.e., the Gi[I/M]Wi method).

Six different Gi(I/M)Wi protocols were tested, each of which was initiated by treating the hiPSCs with 10 μM CHIR for 24 hours; then, cells treated with the Gi(10/0)Wi, Gi(10/1)Wi, Gi(10/2)Wi, Gi(10/3)Wi, Gi(10/4)Wi, and Gi(10/5)Wi protocols were maintained in 0, 1, 2, 3, 4, or 5 μM CHIR, respectively, for 48 hours (i.e., from hour 24 to hour 72 after differentiation was initiated) (Fig. 1b) before Wnt signaling was inhibited via treatment with IWR1 (10 μM) on Day 3. On Day 12 after differentiation was initiated, cTnT expression was significantly more common among cells treated with the Gi(10/2)Wi protocol than among those in any other treatment group (Fig. 1c,d), and two days later, immunofluorescent images and transmission electron micrographs confirmed that the Gi(I/M)Wi-differentiated hiPSC-CMs displayed CM-like characteristics in protein expression (cTnT, myosin light chain 2 [MYL2], sarcomeric α-actinin, connexin 43 [Cx43]) and in the morphology and distribution of myofibrils, Z bands, and mitochondria (Fig. 1e). A beating cell sheet was observed in Gi(10/2)Wi group compared to other groups (Videos S1 and S2).

Perhaps more importantly, our Gi(I/M)Wi method also significantly improved the quantity and reproducibility of cardiomyocyte differentiation in hiPSCs reprogrammed from human dermal fibroblasts (hdiPSCs), which are more resistant than hciPSCs to cardiac differentiation^12^: both the percentage (Fig. 1f) and yield (Fig. 1g) of cTnT^+^ cells were dramatically higher among Gi(10/2)Wi-differentiated cells than among cells differentiated via the traditional GiWi protocol, with little variation observed between hiPSCs of different lineages (hciPSC, hdiPSC) or between hiPSCs that had been passaged 10 or 50 times before differentiation was initiated. We further confirmed our Gi(I/M)Wi method in another genetically modified hciPSCs (Luc ciPSCs)^15^ and found similar boost in percentage of cTnT^+^ cells (Supplemental Fig. 1e). Thus, the Gi(I/M)Wi method substantially improved the efficiency, yield, and reproducibility of hiPSC-CM differentiation in multiple hiPSC lines.

### The low CHIR maintenance dose increases mesendodermal/mesodermal specification while limiting endodermal commitment in differentiating hiPSCs

**Figure 2:**
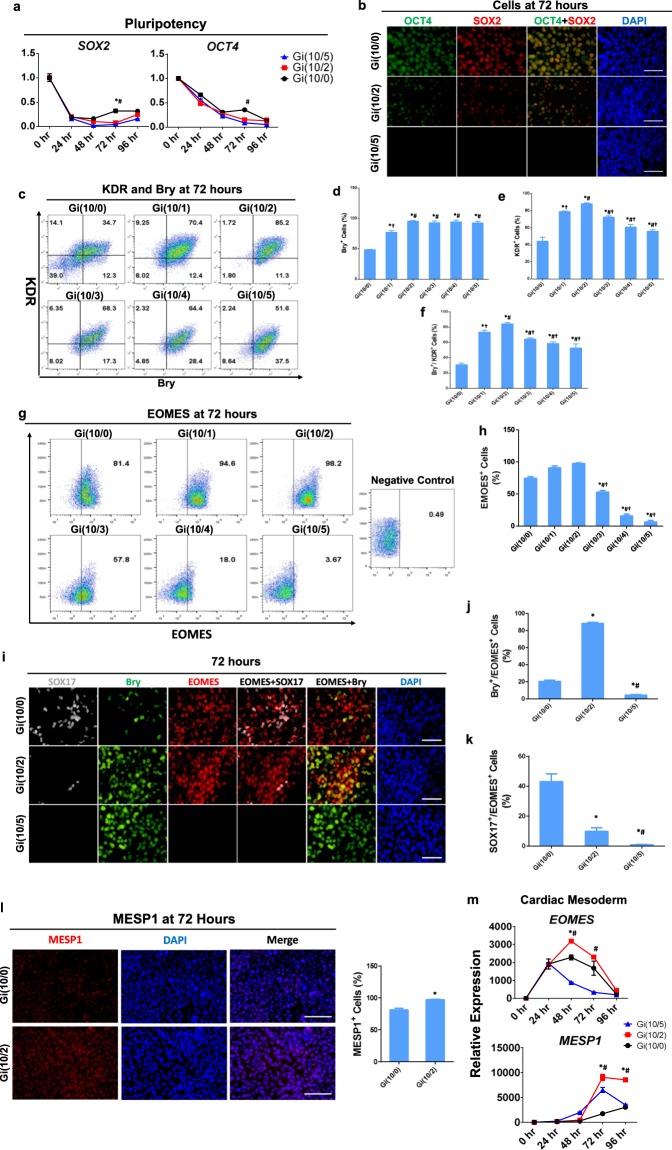
The optimized Gi(I/M)Wi protocol promotes mesendodermal/mesodermal specification and limits endodermal commitment in differentiating hiPSCs. (**a)** mRNA levels of the pluripotency genes SOX2 and OCT4 were quantified via qRT-PCR in Gi(10/0), Gi(10/2), and Gi(10/5) cells at the indicated time points after differentiation was initiated and then normalized to GAPDH mRNA levels and to measurements taken at 0 hr (n = 3 different batches of differentiated cells). *P < 0.05 vs Gi(10/2), ^#^P < 0.05 vs Gi(10/5). (**b**) Gi(10/0), Gi(10/2), and Gi(10/5) cells were collected at Hour 72 of differentiation and immunofluorescently labelled for OCT4 and SOX2 expression; nuclei were counterstained with DAPI (bars = 50 μm). (**c**) Expression of the mesendodermal marker Bry and the mesodermal marker KDR was evaluated in cells from all six treatment groups via flow cytometry at Hour 72 of differentiation; then, the proportion of cells that expressed (**d**) Bry, (**e**) KDR, and **(f**) both Bry and KDR was calculated and presented as a percentage of the total number of cells. *P < 0.05 vs Gi(10/0), ^#^P < 0.05 vs Gi(10/1), ^†^P < 0.05 vs Gi(10/2). (**g**) Expression of the mesendodermal marker EOMES was evaluated in cells from all six treatment groups via flow cytometry at Hour 72 of differentiation; then, (**h**) the proportion of cells that expressed EOMES was calculated and presented as a percentage of the total number of cells. *P < 0.05 vs Gi(10/0), ^#^P < 0.05 vs Gi(10/1), ^†^P < 0.05 vs Gi(10/2). (**i**) Gi(10/0), Gi(10/2), and Gi(10/5) cells were collected at Hour 72 of differentiation in a 96-well plate (3 wells per group), immunofluorescently labelled for Bry and EOMES expression and for expression of the definitive endoderm marker SOX17, and nuclei were counterstained with DAPI (bars = 50 μm); then, the proportion of cells that expressed **(j**) both Bry and EOMES, and (**k**) both SOX17 and EOMES was quantified and presented as a percentage of the total number of cells. *P < 0.05 vs. Gi(10/0). ^#^P < 0.05 vs Gi(10/2). All experiments were repeated three times; 27–30 randomly selected fields (3–4 fields per well) from each group were evaluated. (l) Gi(10/0) and Gi(10/2) cells were collected at Hour 72 of differentiation and immunofluorescently labelled for expression of the mesodermal marker MESP1; nuclei were counterstained with DAPI (bars = 100 μm). The right bar plot showed percentage of MESP1^+^ cells. *P < 0.05; all experiments were repeated three times; 27–30 randomly selected fields (3–4 fields per well) from each group were evaluated. (**m**) mRNA levels of the definitive cardiac mesoderm markers EOMES and MESP1 were quantified via qRT-PCR in Gi(10/0), Gi(10/2), and Gi(10/5) cells at the indicated time points after differentiation was initiated and then normalized to GAPDH mRNA levels and to measurements taken at 0 hr. n = 3 different batches of differentiated cells, *P < 0.05 vs Gi(10/2), ^#^P < 0.05 vs Gi(10/5).

**Figure 3:**
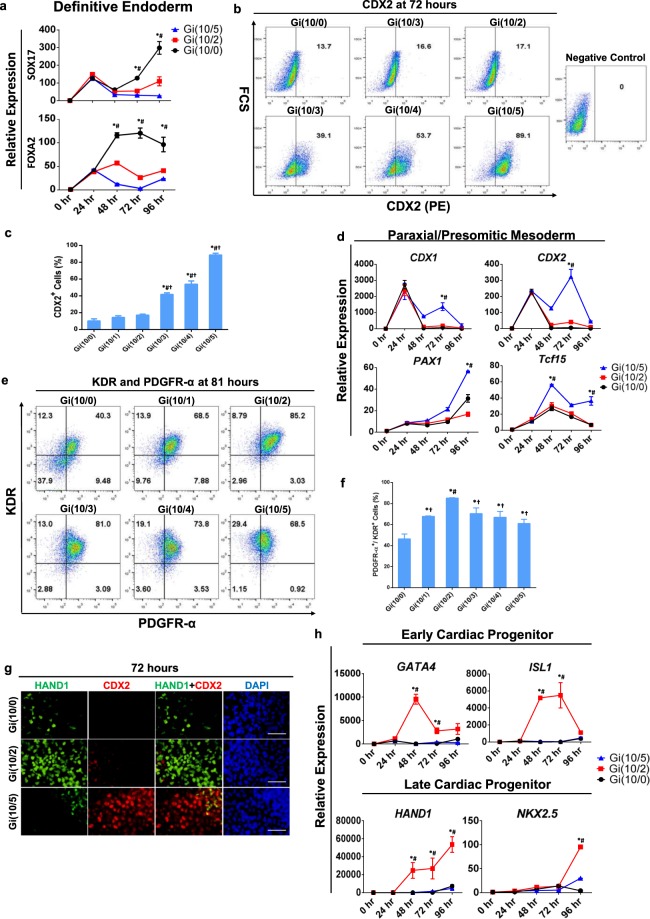
The optimized Gi(I/M)Wi protocol promotes cardiac specification in differentiating hiPSCs. **(a)** mRNA levels of the definitive endodermal genes SOX17 and FOXA2 were quantified via qRT-PCR in Gi(10/0), Gi(10/2), and Gi(10/5) cells at the indicated time points after differentiation was initiated and then normalized to GAPDH mRNA levels and to measurements taken at 0 hr. n = 3 different batches of differentiated cells, *P < 0.05 vs Gi(10/2), ^#^P < 0.05 vs Gi(10/5) **(b)** Expression of the presomitic mesoderm marker CDX2 was evaluated in cells from all six treatment groups via flow cytometry at Hour 72 of differentiation; then, **(c)** the proportion of cells that expressed CDX2 was calculated and presented as a percentage of the total number of cells. *P < 0.05 vs Gi(10/0), ^#^P < 0.05 vs Gi(10/1), ^†^P < 0.05 vs Gi(10/2). **(d)** mRNA levels of the paraxial/presomitic mesoderm markers CDX1, CDX2, PAX1, and Tcf15 were quantified via qRT-PCR in Gi(10/0), Gi(10/2), and Gi(10/5) cells at the indicated time points after differentiation was initiated and then normalized to GAPDH mRNA levels and to measurements taken at 0 hr. *P < 0.05 vs Gi(10/0), ^#^P < 0.05 vs Gi(10/2). **(e)** Expression of the cardiac mesoderm markers KDR and PDGFRα was evaluated in cells from all six treatment groups via flow cytometry at Hour 81 of differentiation; then, **(f)** the proportion of cells that expressed both KDR and PDGFRα was calculated and presented as a percentage of the total number of cells. *P < 0.05 vs Gi(10/0), ^#^P < 0.05 vs Gi(10/1), ^†^P < 0.05 vs Gi(10/2). **(g)** Gi(10/0), Gi(10/2), and Gi(10/5) cells were collected at Hour 72 of differentiation and immunofluorescently labelled for CDX2 expression and for expression of the late CPC marker HAND1; nuclei were counterstained with DAPI **(**bars = 50 μm). **(h)** mRNA levels of the early CPC markers GATA4 and ISL1, and the late CPC markers HAND1 and NKX2.5, were quantified via qRT-PCR in Gi(10/0), Gi(10/2), and Gi(10/5) cells at the indicated time points after differentiation was initiated and then normalized to GAPDH mRNA levels and to measurements taken at 0 hr. *P < 0.05 vs Gi(10/0), ^#^P < 0.05 vs Gi(10/5). All experiments were repeated three times.

Daily measurements of mRNA levels during the first 96 hours of the Gi(I/M)Wi differentiation protocol confirmed that expression of the pluripotency markers SOX2 and OCT4 declined after CHIR treatment was initiated in Gi(10/0)Wi, Gi(10/2)Wi, and Gi(10/5)Wi cells (Fig. 2a); however, pluripotent cells remained more common in Gi(10/0)Wi cells than in the other treatment groups at Hour 72 of differentiation (Fig. 2b). Flow cytometry analyses of mesendodermal marker expression at Hour 72 indicated that Bry^+^ cells became significantly more common (Fig. 2c,d), while EOMES^+^ cell populations also increased (but not significantly) (Fig. 2g,h), as the maintenance CHIR dose rose from 0–2 μM and then remained stable (Bry^+^) or declined significantly (EOMES^+^) at higher maintenance concentrations. Thus, cells that co-expressed the two mesendodermal markers (Bry^+^EOMES^+^) were significantly more common among Gi(10/2)Wi cells than among Gi(10/0)Wi or Gi(10/5)Wi cells (Fig. 2i,j), and subsequent assessments suggested that this increase was accompanied by upregulation of the mesodermal marker MESP1 (Fig. 2l,m). Furthermore, both mRNA (Fig. 3a) and immunofluorescence (Fig. 2i,k, Supplemental Fig. 2a) analyses indicated that the expression of definitive endoderm markers (FOXA2, SOX17) at Hour 72 declined, while cells expressing the presomitic mesoderm marker CDX2 (Fig. 3b,c, Supplemental Fig. 2b) became more common, with increasing maintenance CHIR concentrations. Wnt also contributes to the distribution of human paraxial and lateral mesoderm^16^, and the mRNA levels of CDX2 and other presomitic and paraxial mesoderm markers (CDX1, PAX1, and Tcf15) tended to be higher in Gi(10/5)Wi cells than among Gi(10/0)Wi or Gi(10/2)Wi cells (Fig. 3d). Collectively, these observations suggest that the optimal (2 μM) maintenance CHIR dose maximizes mesendodermal (and perhaps mesodermal) specification while minimizing endodermal and presomitic/paraxial mesodermal commitment.

### The low CHIR maintenance dose increases cardiac-lineage commitment in differentiating hiPSCs

Because CDX2 inhibits cardiac differentiation^17^ and was downregulated by the optimized CHIR maintenance dose, we investigated whether the Gi(I/M)Wi protocol may also have improved cardiac specification during the IWR1 treatment phase. Cells expressing KDR or both KDR and Bry at Hour 72 were most common among the Gi(10/2)Wi group (Fig. 2c,e,f), which is consistent with previous reports that both endocardial and myocardial cells are descended from KDR^+^ cells^18,19^, and that the vascular, hematopoietic, and cardiovascular systems develop from mesodermal progenitor cells that express both KDR^18^ and Bry^20^. Furthermore, although the time-course of expression for KDR, Bry, and PDGFRα (a marker for cardiac mesoderm)^21^ in Gi(10/2)Wi and Gi(10/0)Wi cells was similar—cells that expressed both KDR and Bry declined from Hour 72 to Hour 96 in response to treatment with IWR1 (Supplemental Fig. 3a), while cells expressing both KDR and PDGFRα increased dramatically from Hour 72 to Hour 81 (just nine hours after IWR1 treatment) before declining again at Hour 120 (Supplemental Fig. 3b)—the proportion of KDR^+^Bry^+^ cells was much higher in the Gi(10/2)Wi group (Hour 72: 81.7%, Hour 96: 57.6%) than among Gi(10/0)Wi cells (Hour 72: 34.2%, Hour 96: 8.4%) at each time point, and KDR^+^PDGFRα^+^ cells were significantly more common at Hour 81 in Gi(10/2)Wi cells than in any other treatment group (Fig. 3e,f). HAND1, which plays a key role in CM differentiation and cardiac-specific transcription^22^, was also more commonly expressed among Gi(10/2)Wi cells than among Gi(10/0)Wi or Gi(10/5)Wi cells at Hour 72 (Fig. 3g), and mRNA levels of HAND1 and two early cardiac progenitor cell (CPC) markers, ISL1 and GATA4, were greater in Gi(10/2)Wi than in Gi(10/0)Wi or Gi(10/5)Wi cells as soon as 48 hours after differentiation was initiated (Fig. 3h). Thus, the maintenance CHIR dose may have contributed to cardiac-lineage commitment even before the cells were treated with IWR1. The mRNA levels of HAND1 and another late-CPC marker, NKX2.5, continued to increase in Gi(10/2)Wi cells after treatment with IWR1(Fig. 3h).

### Cardiac mesoderm specification in differentiating hiPSCs is predominantly regulated by the Wnt signaling pathway

Wnt signaling interacts with a number of other pathways, including PI3K/AKT^23^, MAPK/ERK^24^, TGF-β/Activin/Nodal^21^, and BMP2/4^25^, and Western blot analyses indicated that the activated (i.e., phosphorylated) forms of Akt and ERK, as well as smad1/5 and smad2/3, which participate in TGF-β and BMP signaling, were lower on Day 3 of differentiation in Gi(10/2)Wi cells than in the Gi(10/0)Wi group (Supplemental Fig. 4a,b). Thus, we replaced the maintenance CHIR dose with low (1 μM) or high (10 μM) concentrations of pharmacological PI3K/AKT (LY294002), FGF/ERK (SU5402 and PD0325901), BMP4 (LDN193189), and Smad2/3 (SB431542) inhibitors (Supplemental Fig. 4c) to determine whether these pathways also contribute to the improved hiPSC-CM differentiation efficiency achieved with the Gi(I/M)Wi method. Each of the five inhibitors reduced the differentiation efficiency of Gi(10/2)Wi cells by a factor of at least three, and only the low dose of the Smad2/3 inhibitor improved differentiation efficiency in Gi(10/0)Wi cells (Supplemental Fig. 4d). Thus, although moderate declines in smad2/3 phosphorylation and TGF-β/Activin/Nodal signaling may contribute to the improved differentiation efficiency associated with the Gi(I/M)Wi protocol, most of the benefit appears to be directly attributable to the maintenance of Wnt signaling.

### Insulin improves the yield of Gi(I/M)Wi-differentiated hiPSC-CMs by increasing cell survival and proliferation

Gsk inhibitors such as CHIR, BIO, and SB-216763 dose-dependently induce apoptosis in hiPSCs and mouse embryonic stem cells (mESCs)^26,27^, which reduces the yield of hiPSC-CMs or mESC-CMs generated via the traditional GiWi method. However, oxidative stress and apoptosis can be reduced in cultured cells via the addition of insulin^28^, which also participates in the early stages of myocardial development^23^. Thus, we investigated whether the yield of hiPSC-CMs produced via our Gi(I/M)Wi protocol could be enhanced by adding insulin during the high-dose (10 μM) CHIR initiation period. The results from preliminary experiments indicated that when 10 μM CHIR was combined with 0.1, 1, or 10 μg/mL insulin, TUNEL assay showed greatest viability among cells co-treated with 1 μg/mL insulin, while most of the cells co-treated with 10 μg/mL insulin underwent detachment from the plate, unable to processing further (Supplemental Fig. 5a,b). Thus, we evaluated six differentiation protocols in which CHIR was initiated at 10 μM with 1 μg/mL insulin for 24 hours and then maintained at 0, 1, 2, 3, 4, or 5 μM without insulin—the Gi(10 + i/0)Wi, Gi(10 + i/1)Wi, Gi(10 + i/2)Wi, Gi(10 + i/3)Wi, Gi(10 + i/4)Wi, and Gi(10 + i/5)Wi protocols, respectively—for 48 hours before the IWR1 treatment phase began.

**Figure 4:**
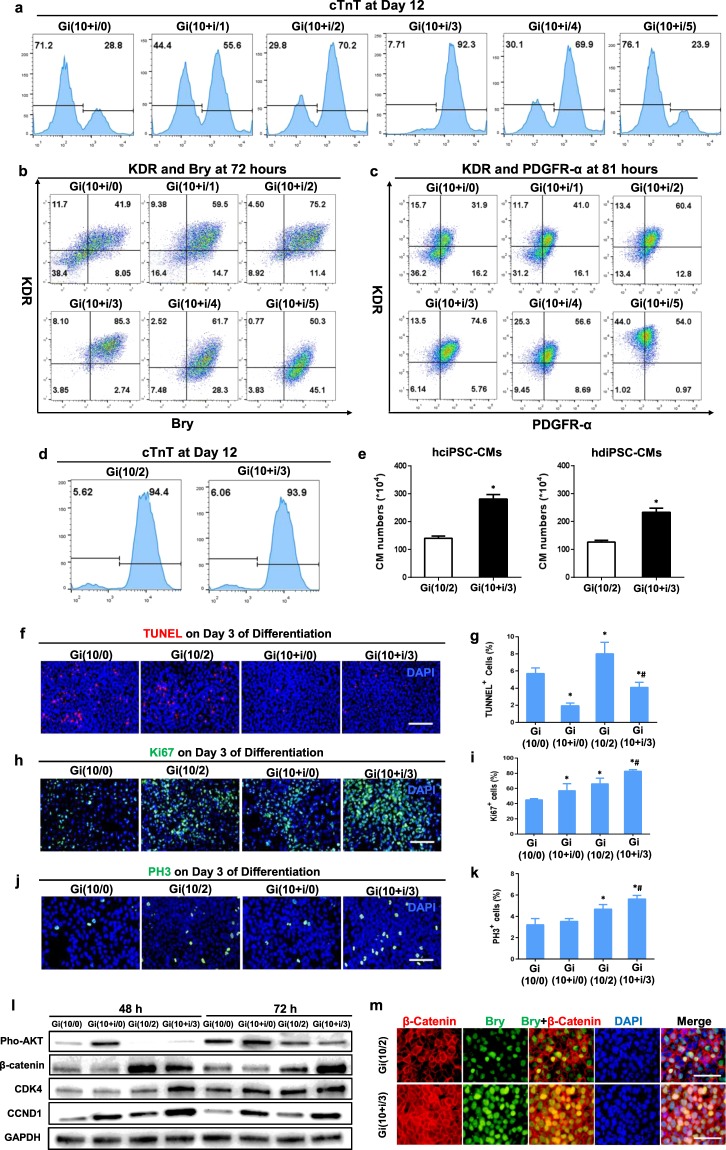
Including insulin during the CHIR initiation phase of the Gi(I/M)Wi protocol improves hiPSC-CM yield by increasing cell survival and proliferation. hiPSCs were treated with 10 μM CHIR and 1 μg/mL insulin for 24 hours; then, cells in the the Gi(10 + i/0), Gi(10 + i/1), Gi(10 + i/2), Gi(10 + i/3), Gi(10 + i/4), and Gi(10 + i/5) groups were maintained in 0, 1, 2, 3, 4, or 5 μM CHIR (without insulin), respectively, for 48 hours before IWR1 (10 μM) treatment began on Day 3. **(a)** cTnT expression in cells from all six treatment groups was evaluated via flow cytometry 12 days after differentiation was initiated. **(b,c)** The expression of **(b)** KDR and Bry and **(c)** KDR and PDGFRα was evaluated via flow cytometry 72 hours and 81 hours, respectively, after differentiation was initiated in cells from all six treatment groups. **(d)** Twelve days after differentiation was initiated, cTnT expression was evaluated via flow cytometry in cells from the Gi(10/2) group (Fig. 1b) and in cells from the Gi(10 + i/3) group. **(e)** hciPSCs and hdiPSCs were differentiated via the Gi(I/M)Wi protocol; cells in the Gi(10/2) group were treated with CHIR initial and maintenance doses of 10 μM (without insulin) and 2 μM, and cells in the Gi(10 + i/3) group were treated with CHIR initial and maintenance doses of 10 μM with 1 μg/mL insulin and 3 μM; then, the total number of CMs in each group was determined via flow cytometry analyses of cTnT expression. P < 0.05 vs Gi(10/2). (**f**) Cells from the Gi(10/0) and Gi(10/2) groups (Fig. 1b) and from the Gi(10 + i/0) and Gi(10 + i/3) groups were TUNEL-stained, and nuclei were counter-stained with DAPI (bar = 100 μm); then, (**g**) the number of TUNEL^+^ cells was determined and expressed as a percentage of the total number of cells (n = 3 different batches of differentiated cells). *P < 0.05 vs Gi(10/0), ^#^P < 0.05 vs Gi(10/2). 27 fields (3 fields per well) from each group were evaluated. (h) Gi(10/0), Gi(10/2), Gi(10 + i/0), and Gi(10 + i/3) cells were immunofluorescently stained for expression of the proliferation marker Ki67 and nuclei were counterstained with DAPI (bar = 100 μm); then, (**i**) the number of Ki67^+^ cells was determined and expressed as a percentage of the total number of cells (n = 3 different batches of differentiated cells). *P < 0.05 vs. Gi(10/0), ^#^P < 0.05 vs Gi(10/2). (**j**) Gi(10/0), Gi(10/2), Gi(10 + i/0), and Gi(10 + i/3) cells were immunofluorescently stained for expression of the proliferation marker PH3 and nuclei were counterstained with DAPI (bar = 100 μm); then, (**k**) the number of PH3^+^ cells was determined and expressed as a percentage of the total number of cells. *P < 0.05 vs. Gi(10/0), ^#^P < 0.05 vs Gi(10/2). (**l**) Protein levels of phosphorylated AKT (Pho-AKT), β-catenin, and the cell-cycle regulatory molecules CDK4 and CCND1 were evaluated via Western blot in Gi(10/0), Gi(10 + i/0), Gi(10/2), and Gi(10 + i/3) cells 48 and 72 hours after differentiation was initiated; GAPDH levels were also determined to confirm equal loading. The blots were collected from different gels with no additional crop process. Different blots were separated by white space. (**m**) Gi(10/2) and Gi(10 + i/3) cells were collected at 72 hours of differentiation and immunofluorescently stained for the expression of Bry and β-catenin; nuclei were counterstained with DAPI (bar = 50 μm). All experiments were repeated three times; 27–30 randomly selected fields (3–4 fields per well) from each group were evaluated.

When 1 μg/mL insulin was included during the CHIR initiation phase, the greatest differentiation efficiency (Fig. 4a, Videos S3 and S4), as well as the highest proportion of KDR^+^Bry^+^ (Fig. 4b) and KDR^+^PDGFRα^+^ (Fig. 4c) cells at Hours 72 and 81 (respectively), was obtained with a maintenance CHIR concentration of 3 μM. Furthermore, although the differentiation efficiency was no better when insulin was added during the CHIR initiation period of Gi(10 + i/3)Wi cells than without insulin in Gi(10/2)Wi cells (~94% each) (Fig. 4d), the Gi(10 + i/3)Wi protocol produced approximately twice as many hiPSC-CMs from both hciPSCs and hdiPSCs (Fig. 4e). Apoptotic (i.e., TUNEL^+^) cells were also significantly less common (Fig. 4f,g), while cells expressing the proliferation markers Ki67 (Fig. 4h,i) and phosphorylated histone 3 (PH3) (Fig. 4j,k) were significantly more common, among Gi(10 + i/3)Wi cells than Gi(10/2)Wi cells, and Western-blot (Fig. 4l) and/or immunofluorescence imaging (Fig. 4m) analyses suggested that the addition of insulin increased Akt activation and Wnt signaling (i.e., β-catenin levels), as well as the expression of two cell-cycle regulatory molecules (CDK4 and CCND1). Collectively, these observations indicate that the inclusion of insulin during the CHIR initiation phase increases the yield of differentiated hiPSC-CMs by reducing apoptosis^29^ and promoting proliferation^30^.

### The Gi(I/M)Wi protocol efficiently induces hiPSC-CM differentiation in suspended cells

**Figure 5:**
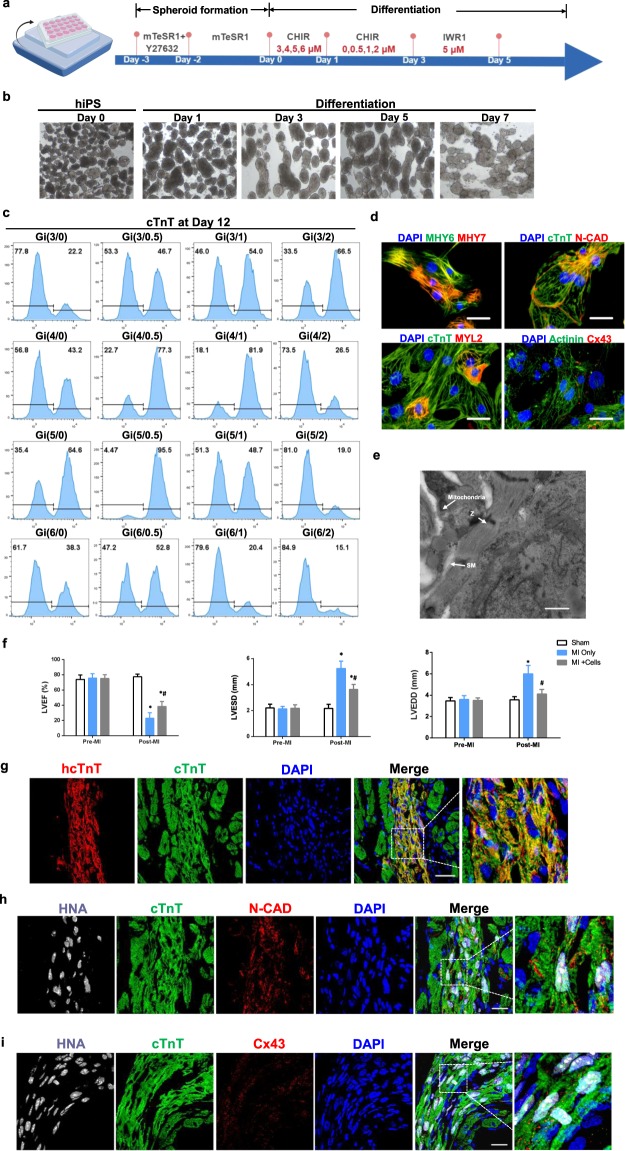
The Gi(I/M)Wi protocol can be adapted to efficiently induce hiPSC-CM differentiation in suspended cells. **(a)** Application of the Gi(I/M)Wi protocol to a cell-suspension system is illustrated schematically. The hiPSCs were allowed to form spheroids for 3 days before differentiation was initiated on Day 0, and 16 combinations of CHIR initiation (3, 4, 5, or 6 μM) and maintenance (0, 0.5, 1, or 2 μM) doses were evaluated. **(b)** Representative bright-field images of differentiating spheroids at the indicated time points. **(c–e)** Twelve days after differentiation was initiated **(c)** cTnT expression was evaluated in cells from all 16 Gi(I/M)Wi treatment groups via flow cytometry (n = 3 different batches of differentiated cells); and **(d,e)** cells from the Gi(5/0.5) group were **(d)** immunofluorescently stained for the expression of MYH6, MYH7, cTnT, N-cadherin (N-CAD), MYL2, α-actinin (Actinin) and Cx43 (bars = 40 μm), and **(e)** imaged via transmission electron microscopy (Z: Z-band, SM: sarcomere; bars = 2 μm). **(f,g)** Myocardial infarction (MI) was surgically induced in mice; then, the animals were administered 1 million hiPSC-CMs (MI + Cells) or 25 μl of PBS (MI Only). The hiPSC-CMs had been differentiated via the Gi(I/M)Wi protocol in a cell-suspension system, and a third group of animals (the Sham group) underwent sham surgery and recovered without either experimental treatment. (**f**) Cardiac function was evaluated via echocardiographic assessments of LVEF, LVESD, and LVEDD before MI and 30 days afterward. N = 7 in sham and MI only group. N = 8 in MI + cells group. *P < 0.05 vs Sham, ^#^P < 0.05 vs MI only. (**g–i)** Heart tissues were harvested from MI + Cells animals 30 days after MI and immunofluorescently stained for the expression of **(g)** human cTnT (hcTnT), cTnT; **(h)** HNA, cTnT and N-CAD; **(i)** HNA, cTnT and Cx43; nuclei were counterstained with DAPI (bars = 20 μm).

Because the monolayer culture system used to develop our Gi(I/M)Wi hiPSC-CM differentiation protocol is not well-suited for the large-scale production of hiPSC-CMs, we investigated whether our method could be adapted to a suspension culture system. The results from preliminary experiments indicated that differentiation was negligible when CHIR treatment was initiated at a concentration of 3 μM or less, and that concentrations greater than 6 μM led to a precipitous decline in cell survival (data not shown); thus, we evaluated 16 combinations of CHIR initiation (3, 4, 5, or 6 μM for 24 hours) and maintenance (0, 0.5, 1, or 2 μM for 48 hours) concentrations. hiPSCs were seeded into low-binding plates and formed spheroids of undifferentiated cells that were allowed to grow for three days before the CHIR treatment phase began on Day 0; then, after the 72-hour CHIR treatment period, IWR1 treatment was administered at 5 μM from Day 3 until Day 5 (Fig. 5a,b), and differentiation efficiency was evaluated via flow-cytometry analyses of cTnT expression on Day 12. Maximum efficiency (95.5%) was achieved when CHIR was initiated at 5 μM and maintained at 0.5 μM (Fig. 5c), and subsequent analyses confirmed that the differentiated hiPSC-CMs displayed CM-like characteristics in patterns of protein expression (myosin heavy chain 6 and 7 [MYH6, MYH7], cTnT, N-cadherin [N-CAD], MYL2, α-actinin, and Cx43) (Fig. 5d) and in the morphology and distribution of Z-bands and mitochondria (Fig. 5e). The cardiomyocytes cultured in suspension culture showed spontaneously beating after differentiation (Videos S5 and S6). In order to test whether insulin treatment could be adapted into suspension culture, we further evaluated the differentiation efficiency using flow-cytometry analyses of cTnT expression on Day 12 for insulin treated spheroid **(**Supplemental Fig. 5c). In accordance to monolayer culture, the highest percentages of cTnT expression were achieved with higher maintenance CHIR concentration (1 μM) when compared to untreated group (0.5 μM). Similarly, the optimal concentration of insulin is 1 μg/ml, leading to the best outcome of hiPSC-CM differentiation.

Since *in-vivo* studies of transplanted CMs have rarely been conducted with cells generated in a suspension system, we also evaluated the hiPSC-CMs produced via our Gi(5/0.5)Wi suspension method in a mouse model of myocardial infarction (MI). One million cells or an equivalent volume of the delivery vehicle (25 μl of PBS) were injected into the infarcted heart of each mouse, and four weeks later, echocardiographic assessments of left ventricular (LV) ejection fraction (EF), end-systolic diameter (ESD) and end-diastolic diameter (EDD) were significantly better in hiPSC-CM-treated than in vehicle-treated animals (Fig. 5f). Furthermore, the magnitude of improvement was consistent with previous studies of monolayer-generated hiPSC-CMs in the same animal model^15^, and immunofluorescence analyses of human cTnT (hcTnT), N-CAD, CX43, and human nuclear antigen (HNA) expression identified transplanted hiPSC-CMs in the border zone of infarction (Fig. 5g–i).

## Discussion

Human induced-pluripotent stem cells (hiPSCs) can be differentiated into cardiomyocytes (CMs) via treatment with Gsk3β (e.g. CHIR99021) and Wnt (IWR1) inhibitors (i.e., the GiWi protocol); however, generation of large number hiPSC-CMs with high efficiency and purity has been a limitation for its widespread application. Here, we introduce a modified Gi(I/M)Wi protocol in which CHIR99021 treatment is initiated (I) at a high dose for 24 hours and then maintained (M) at a lower dose until IWR1 treatment begins 48 hours later. For hiPSC monolayers, optimal efficiency (>90%) was achieved with I/M doses of 10 μM/2 μM CHIR99021, that the hiPSC-CM yield could be increased 2-fold by including insulin during the CHIR99021 initiation phase and increasing the maintenance CHIR99021 dose to 3 μM, and that I/M doses of 5 μM/0.5 μM CHIR99021 produced maximum differentiation efficiency in suspended cells. Mechanistically, over-activation of Wnt leads to presomitic mesoderm differentiation and insufficient Wnt will form definitive endoderm. Thus, the Gi(I/M)Wi hiPSC-CM differentiation protocol can be fine-tuned to maximize differentiation efficiency for each hiPSC line and culture condition.

Wnt’s involvement in mammalian embryonic development begins as early as the gastrulation phase, when interactions between the Wnt proteins and their inhibitors establish a gradient of Wnt/β-catenin activity that is instrumental for formation of the anteroposterior and dorsoventral axes^31^. The role of Wnt in cardiac tissue formation and cardiogenesis^7,32,33^, is recapitulated by the GiWi protocol to drive the differentiation of hiPSCs into CMs^34^; however, the efficiency and yield of the conventional GiWi protocol can vary substantially across hiPSC lines^4^, and methods for customizing the protocol to optimize differentiation for any individual hiPSC population had yet to be investigated. Here, we present a modified version of the GiWi protocol, Gi(I/M)Wi, in which the Wnt activation (i.e., Gi) period is conducted in two phases: Wnt activity is induced with a high initial (I) dose of the Gsk3β inhibitor CHIR for 24 hours and then maintained (M) with a lower CHIR concentration for the ensuing 48 hours before the Wnt inhibition phase begins. The flexibility provided by the CHIR initiation and maintenance (I/M) doses enables the magnitude of Wnt activity to be fine-tuned for each hiPSC line and to accommodate other factors that may improve hiPSC-CM differentiation, such as insulin, which promotes cell viability and proliferation but impedes Wnt signaling (also see Fig. 4i, β-catenin Gi[10/0] vs Gi[10 + /0])^35^. For example, our results indicate that under 2-dimensional culture conditions, the optimal efficiency for differentiating cardiac-lineage hiPSCs into CMs is achieved with a maintenance CHIR dose of 2 μM, and equivalent efficiency, but an approximately 2-fold greater yield, can be produced by including insulin during the CHIR initiation phase and increasing the maintenance CHIR dose to 3 μM. The minor modification of CHIR concentrate is largely attributed to the potential crosstalk between insulin, Wnt/β-catenin and AKT pathways^35,36^ and aims to balance their opposite effects on hiPSC-CM differentiation. 3 μM of CHIR could restore the impaired activity of Wnt/β-catenin and elevated phosphate AKT under treatment of insulin after 48 hours of differentiation (Fig. 4i). The Gi(I/M)Wi protocol is also compatible with the high-throughput (e.g., cell-suspension) systems needed to produce the large number of cells required for cell therapy (~1 billion per patient)^37^ and cardiac tissue engineering^38^.

**Figure 6:**
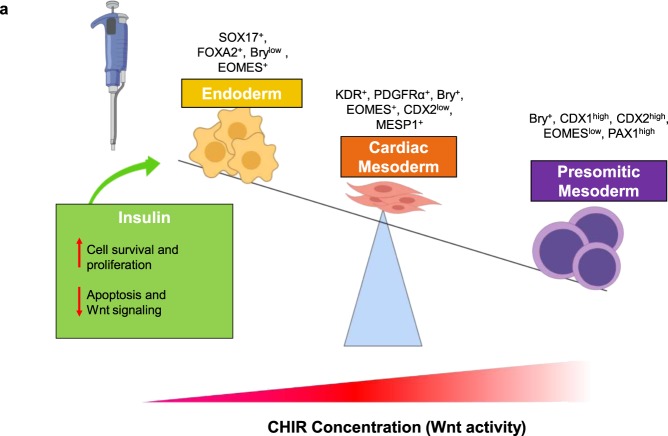
The role of Wnt signaling in hiPSC-CM differentiation. Wnt signaling is required to reduce pluripotency, limit endodermal specification (SOX17, FOXA2), and promote mesendodermal (Bry, EOMES) commitment in differentiating hiPSCs; however, excessive Wnt activation directs the cells toward the paraxial/presomitic mesoderm lineage (CDX1, CDX2, PAX1). The Gi(I/M)Wi protocol enables researchers to balance these antagonistic effects of Wnt activity for maximum cardiac mesodermal specification and hiPSC-CM yield by adjusting the CHIR initiation and maintenance doses for different hiPSC lines (e.g., hciPSCs or hdiPSCs) and culture systems (e.g., monolayer or suspension), or to incorporate other factors such as insulin, which increases cell survival and proliferation but partially limits Wnt activity.

Previous reports have shown that Wnt signaling downregulates pluripotency genes^39^ and upregulates endodermal genes (e.g., SOX17 and FOXA2)^40^ when pluripotency gene expression is insufficiently suppressed. These results are consistent with our observation that cells in the Gi(10/0)Wi group continued to express OCT4 and SOX2 during the CHIR maintenance phase and displayed the highest levels of SOX17 and FOXA2 expression as early as 48 or 72 hours after differentiation was initiated. Furthermore, Wnt signaling is known to initiate and/or sustain the expression of CDX1^41^ and CDX2^42^, which inhibit cardiac specification by promoting the hematopoietic or presomitic mesodermal lineages^17,43^, and our results indicate that as the maintenance CHIR dose was increased from 2 to 5 μM, the expression of presomitic and paraxial markers (CDX1, CDX2, PAX1, and Tcf15) also increased, while markers for cardiac-lineage specification declined. We also show that in a suspension system, differentiation efficiency tended to improve when higher CHIR initiation doses were paired with lower maintenance doses (and vice-versa). Collectively, these observations suggest that the Gi(I/M)Wi protocol optimizes hiPSC-CM differentiation by balancing the level and duration of Wnt pathway activation to minimize both endodermal and presomitic-mesodermal commitment (Fig. 6). Notably, the optimal concentrations for both the initiation and maintenance phases of CHIR treatment were lower when the cells were differentiated in a suspension system rather than in monolayers, perhaps because the surfaces of the suspended cells were more exposed to the culture medium.

In conclusion, the novel findings of current study demonstrate an important temporal factor of Wnt activation in regulation of hiPSC-CM differentiation efficiency and consistency and provide a high-throughput and stable method for hiPSC-CM production.

## Methods

All protocols and experimental procedures involving animals were approved by the Institutional Animal Care and Use Committee (IACUC) of the University of Alabama at Birmingham and performed in accordance with the National Institutes of Health Guide for the Care and Use of Laboratory Animals (National Institutes of Health publication No. 85-23).

### hiPSC culture

Three iPS cell lines used in this study, gifted from Dr. Liying Zhang, were generated from either human cardiac fibroblast or dermal fibroblast as previously described^12^. The fibroblasts were collected from 3 patients (2 females, 1 male) with protocols approved by the University of Minnesota Human Subjects Research Institutional Review Board. iPSCs were generated with Sendai Virus mediated non-integration method and fully characterized^12^. hiPSCs were cultured in mTeSR1 medium (Stemcell technologies; 85850) with daily medium changes on Matrigel-coated (Fisher scientific; CB356253) 6-well plates and passaged in a 1:6 ratio after reaching 90% confluency (every 3-4 days). For passaging, the medium was aspirated, and the cells were washed with phosphate-buffered saline (PBS) and dissociated with Accutase (Fisher scientific; MT25058CI) (500 μL per well for 5–7 min at 37 °C); then, the disassociation reaction was neutralized with mTeSR1 medium supplemented with 5 μM Y-27632 (Stemcell technologies; 72304), and the mixture was centrifuged for 3 min at 1100 rpm. The supernatant was removed, and the cells were resuspended in mTeSR1 medium supplemented with 5 μM Y-27632 and distributed into a new Matrigel-coated 6-well plate.

### hiPSC-CM differentiation

The Gi(I/M)Wi differentiation protocol was conducted as described in the Results section with the reagents listed in Supplemental Table 1. hiPSCs were seeded into each well of a Matrigel-coated 6-well plate (0.2 million/well). When monolayers of hiPSCs are 85–90% confluent, cardiac differentiation were performed. For the cell-suspension system, the hiPSCs were dissociated, suspended in mTeSR1 medium supplemented with 5 μM Y-27632, transferred into a low-binding 24-well plate (MBL International; NCP-LH24-2) (1 × 10^6^ cells/mL, 1 mL/well), and maintained in a horizontal shaker for 24 hr. Aggregates of cells were typically observed after 24 hours of shaking, and the medium was replaced with mTeSR1 only for 48 hr before differentiation was initiated.

CHIR99021 (Stemcell technologies; 72054) and IWR1 (Stemcell technologies; 72562) were added at the indicated concentrations and time points in RPMI1640/B27 without insulin (Fisher Scientific; 11-875-119 and A1895601). After the 48 hr IWR1 treatment period, the medium was replaced with RPMI/B27 minus insulin for 48 hours (i.e., from Day 5 to Day 7); then, the cells were maintained in RPMI/B27 with insulin (Fisher Scientific; 11-875-119 and 17-504-044) with medium changes every 2-3 days. Beating cardiomyocytes were typically observed 7–10 days after differentiation was initiated.

### Flow cytometry

Cells were disassociated with TrypLE Express Enzyme (Fisher Scientific; 12-605-010) for 5–10 min at 37 °C; then, the dissociation reaction was neutralized with RPMI20 (RPMI1640 + 20% fetal bovine serum [FBS]) medium. When live cells were not required, cells were fixed^12^ with 1% paraformaldehyde (PFA) for 10 min in a 37 °C water bath, permeabilized in cooled 90% acetone for 3 min at −20 °C, washed three times with fluorescence-activated cell sorter (FACS) buffer (0.5% bovine serum albumin [BSA] and 0.1% Triton X-100 in PBS), incubated with primary antibodies (Supplemental Table 1) overnight at 4 °C, washed three times with FACS buffer, incubated with secondary antibodies at room temperature for 20 min, washed with FACS buffer three times, and resuspended in 200 μL FACS buffer. When marker expression could only be conducted with live cells, the cells were incubated with fluorescence-conjugated primary antibodies diluted in live-cell FACS buffer (1% BSA, 10 μM Y-27632 in PBS) for 1 hr at room temperature, washed, and resuspended in live-cell FACS buffer. Flow cytometry was performed with a BD LSR II analyzer, and the data was analyzed with FlowJo Version 8.8.7 software (FlowJo, LLC).

### Mouse model of myocardial infarction and cell administration

All protocols and experimental procedures involving animals were approved by the Institutional Animal Care and Use Committee (IACUC) of the University of Alabama at Birmingham and performed in accordance with the National Institutes of Health Guide for the Care and Use of Laboratory Animals (National Institutes of Health publication No. 85-23). Myocardial infarction (MI) was induced in 12-week-old NOD/SCID mice (20–25 g bodyweight; The Jackson Laboratory) as described previously^44^. Briefly, mice were anesthetized with inhaled isoflurane (1.5–2%), orally intubated, and ventilated with a MINIVENT mouse ventilator (Type 845; tidal volume: 100–150 μL, respiratory rate: 100–150 bpm). An incision was made in the intercostal space of the left thorax, and the left anterior descending coronary artery (LAD) was ligated with an 8–0 nonabsorbable suture near the origin of the pulmonary outflow tract and the left atrial margin. Immediately after the LAD ligation, the cells were injected into the myocardium at three sites (1 × 10^6^ cells/animal, 3.3 × 10^5^ cells/site); one site was located in the infarcted region and two were in the peri-infarct region. After cell administration, the incision was sutured, and the mice were placed on a warm hot pad until conscious. Buprenorphine (0.1 mg/kg every 12 hours for 3 days) and ibuprofen (5 mg/kg every 12 hours for 1 day) were administered intraperitoneally for post-operative pain control.

### Echocardiography

Mice were anesthetized with 2% isoflurane and heart rate was monitored to maintain approximately 400–500 beats/min. After shaving the chest hair, the mice were placed on a warm pad to keep the body temperature at around 37 °C. Echocardiography was performed in a commercially available echocardiographic system equipped with a 18–38 MHz phased array transducer (Vevo 2100, VisualSonics Inc). Place the transducer along the long axis of the LV and guide it to the right side of the mouse neck to obtain a parasternal left ventricle (LV) long-axis image. Next, the transducer is rotated by 90° to obtain a two-dimensional LV short-axis image. LV ejection fraction (LVEF), LV end-systolic dimension (LVESD) and LV end-diastolic dimension (LVEDD) were calculated using the Vevo analysis software.

### Immunohistochemistry

Cells and tissue sections were fixed with 4% PFA for 10 min at room temperature and permeabilized in cooled acetone for 3 min at −20 °C, washed with 0.1% TWEEN20 in PBS (PBST) three times, blocked with 5% donkey serum (Sigma-Aldrich; D9663) for 30 min at room temperature, incubated with primary antibodies (Supplemental Table 1) overnight at 4 °C, washed three times with PBST, incubated with secondary antibodies for 30 min at room temperature, and mounted with DAPI-containing mounting medium (Vector Laboratories; H-1200). Images were obtained with an Olympus IX83 fluorescence microscope, and data analysis was performed with Image J software.

### Western blot

Cells were lysed with Protein Extraction Reagent (Fisher Scientific; 78510) supplemented with protease and phosphatase inhibitors (Fisher Scientific; 78442); then, the cell extracts were subjected to electrophoresis on 10% Mini-PROTEAN TGXTM Precast Gels (BIO-RAD; 4568033), and the separated proteins were transferred to a nitrocellulose membrane (BIO-RAD; 1704270) by using a Trans-Blot Turbo transfer apparatus (BIO-RAD). The membrane was incubated with 5% non-fat milk (BIO-RAD; 1706404) in PBST for 60 min at room temperature and with primary antibodies (Supplemental Table 1) overnight at 4 °C; then, the membrane was washed three times with TBST, incubated with horseradish peroxidase-conjugated secondary antibodies (1:3000 dilution) for 30 min at room temperature, and washed three more times with TBST. Protein signals were developed with HRP-substrate (MILLIPORE; WBKLS0500) and images were acquired with a ChemiDocTM Imaging System (BIO-RAD).

### Quantitative RT-PCR (qRT-PCR)

Total RNA was extracted with TRIzol reagent (Fisher Scientific; 15596-018) and treated with DNase (Sigma-Aldrich; AMPD1-1KT); then, the quantity and quality of RNA were evaluate with a Nanodrop 2000 spectrophotometer (Thermo Scientific). Reverse transcription was performed with TaqMan Reverse Transcription Reagents (Fisher Scientific; 4304134), and RT-PCR was performed by using Fast SYBR Green Master Mix (Fisher Scientific; K0251) and the primer sequences listed in Supplemental Table 2 on a Quantstudio Real-time PCR system (Fisher Scientific). GAPDH was used as an endogenous control, and mRNA levels were calculated via the ΔΔCT method.

### Statistical analysis

Data are presented as mean ± SEM of replicate experiments. Differences between two or more groups were evaluated for significance via the Student’s t test or one-way analysis of variance (ANOVA), and a p value of less than 0.05 was considered statistically significant.

## Supplementary information

Supplementary Information

Supplementary Video S1

Supplementary Video S2

Supplementary Video S3

Supplementary Video S4

Supplementary Video S5

Supplementary Video S6

## Data Availability

All data related to the research conducted for this manuscript are available upon request from the authors.
