# Supplementary material for: Deciphering Role of Wnt Signalling in Cardiac Mesoderm and Cardiomyocyte Differentiation from Human iPSCs: Four-dimensional control of Wnt pathway for hiPSC-CMs differentiation
Source: Sci Rep. 2019 Dec 18;9:19389. doi: 10.1038/s41598-019-55620-x (PMC6920374; doi:10.1038/s41598-019-55620-x)
Supplement: Supplementary file 1 — Supplementary Information [file 41598_2019_55620_MOESM1_ESM.pdf]

Deciphering Role of Wnt Signalling in Cardiac Mesoderm and Cardiomyocyte Differentiation from Human iPSCs: Four-dimensional control of Wnt pathway for hiPSC-CMs differentiation

Meng Zhao[1#], Yawen Tang[1#], Yang Zhou[1], Jianyi Zhang[1*]
[1]Department of Biomedical Engineering, School of Medicine, School of Engineering, University of Alabama at Birmingham, Birmingham, AL35233, USA.

#These authors contributed equally: Meng Zhao, Yawen Tang.

Correspondence and requests for materials should be addressed to J.Z. (email: jayzhang@uab.edu)

Supplemental Figure 1

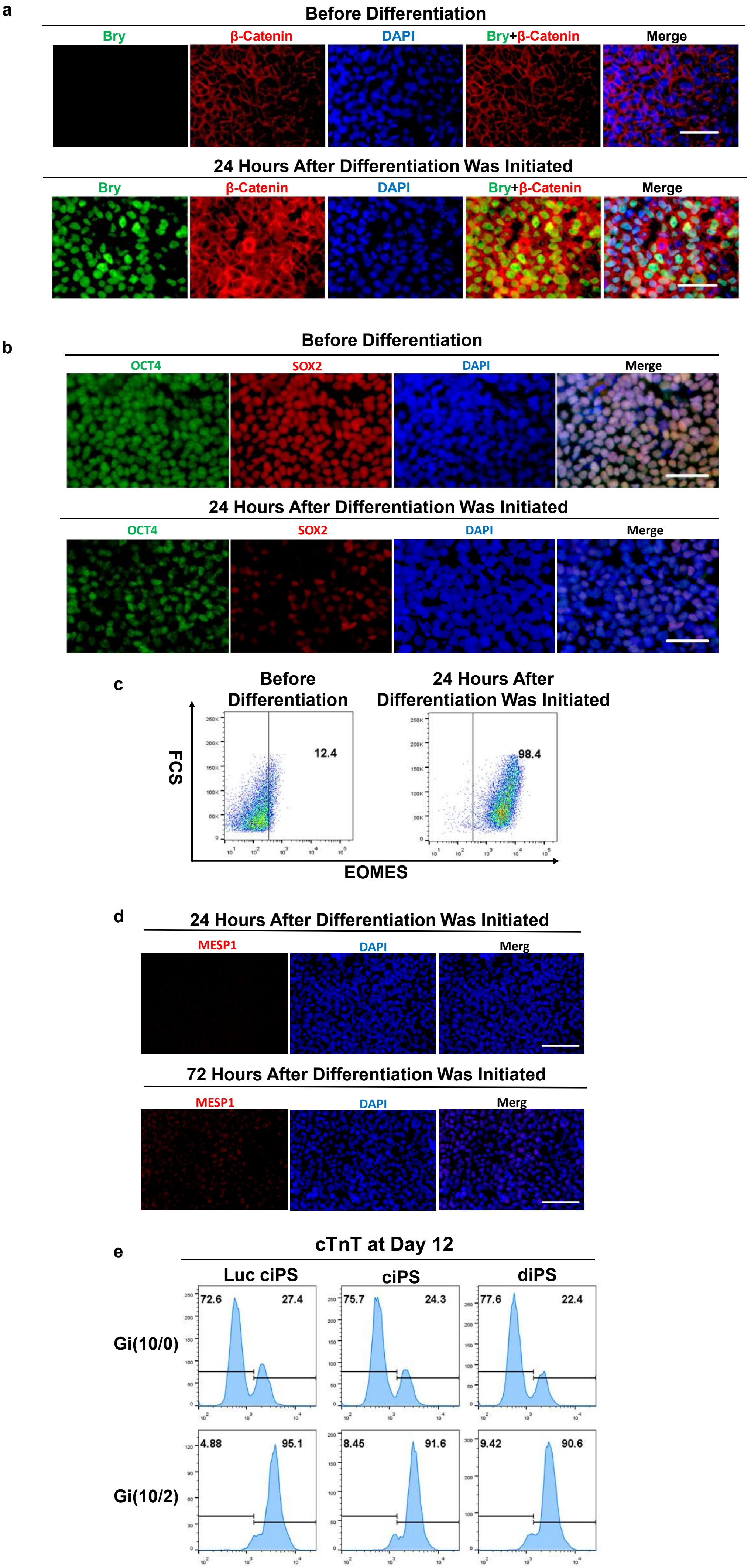

Supplemental Figure 2

a

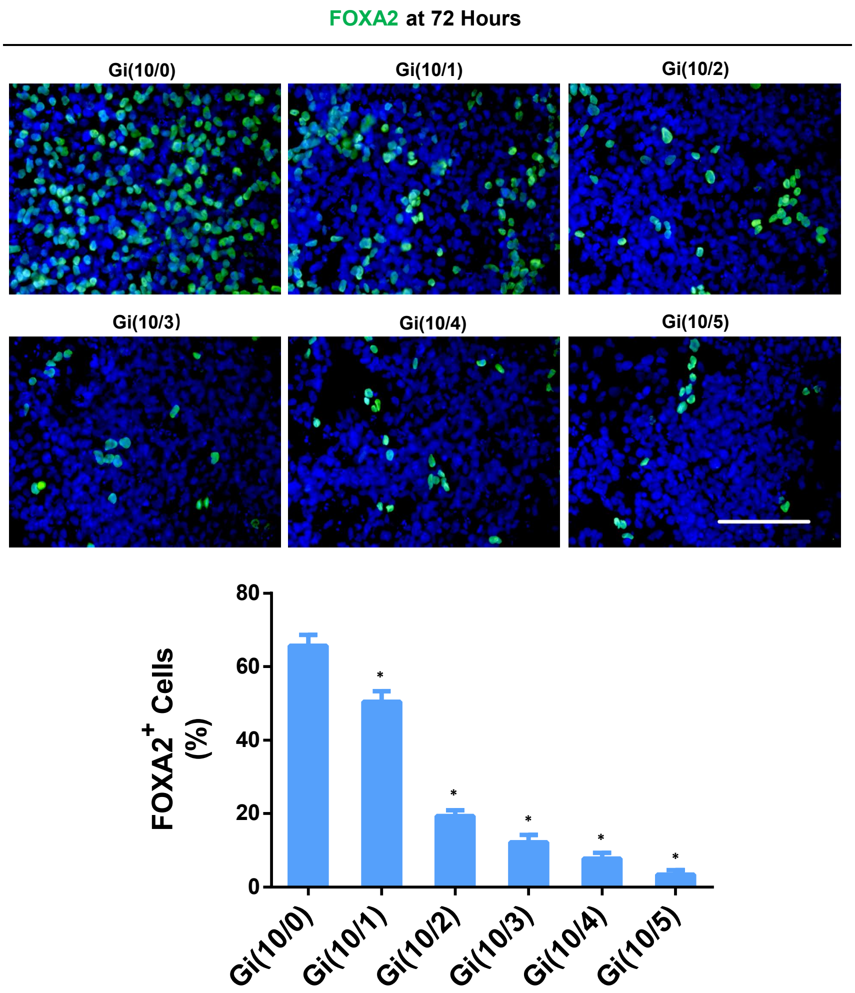

b

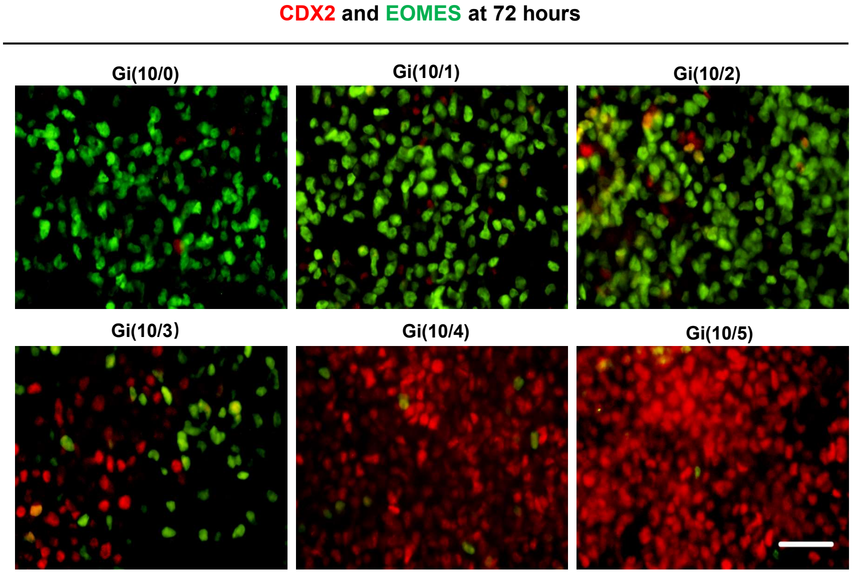

Supplemental Figure 3

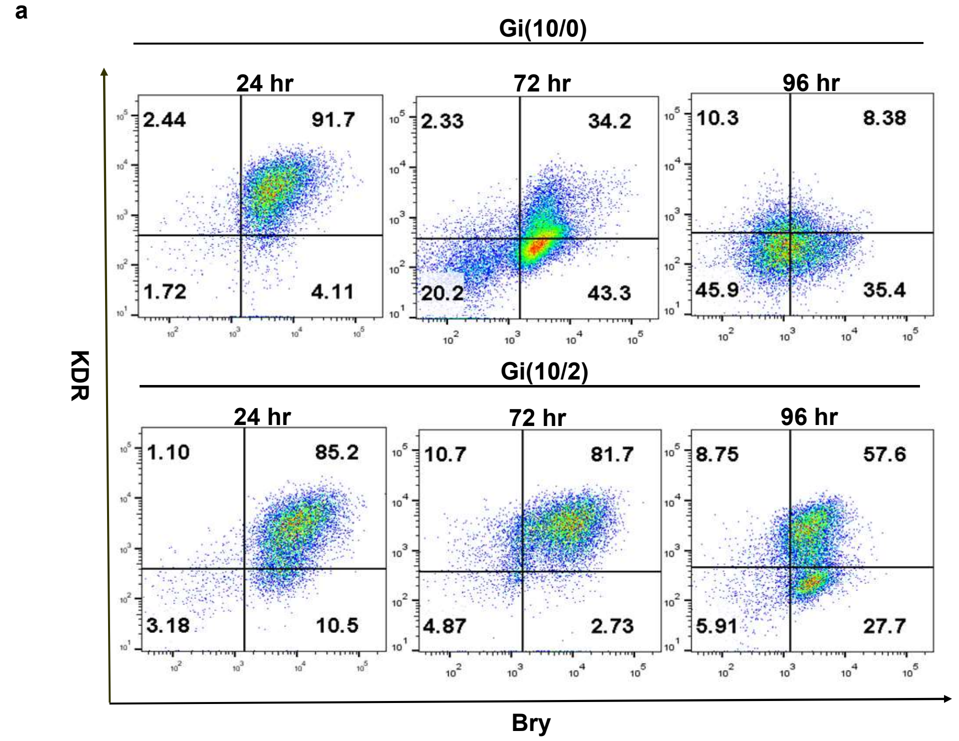

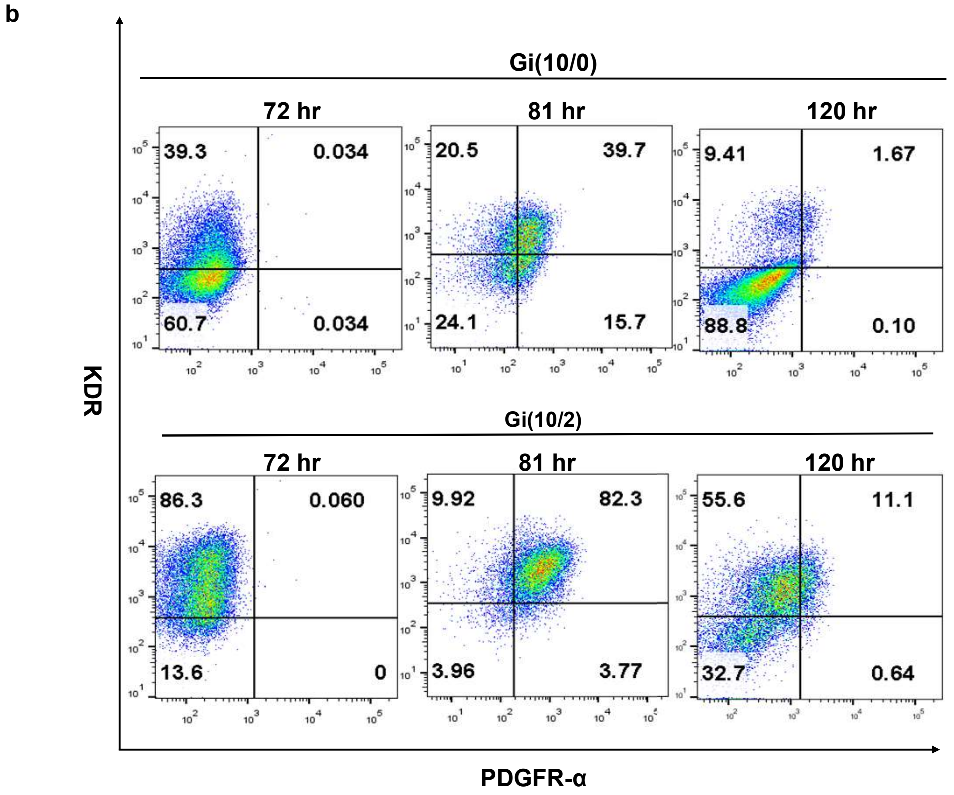

Supplemental Figure 4

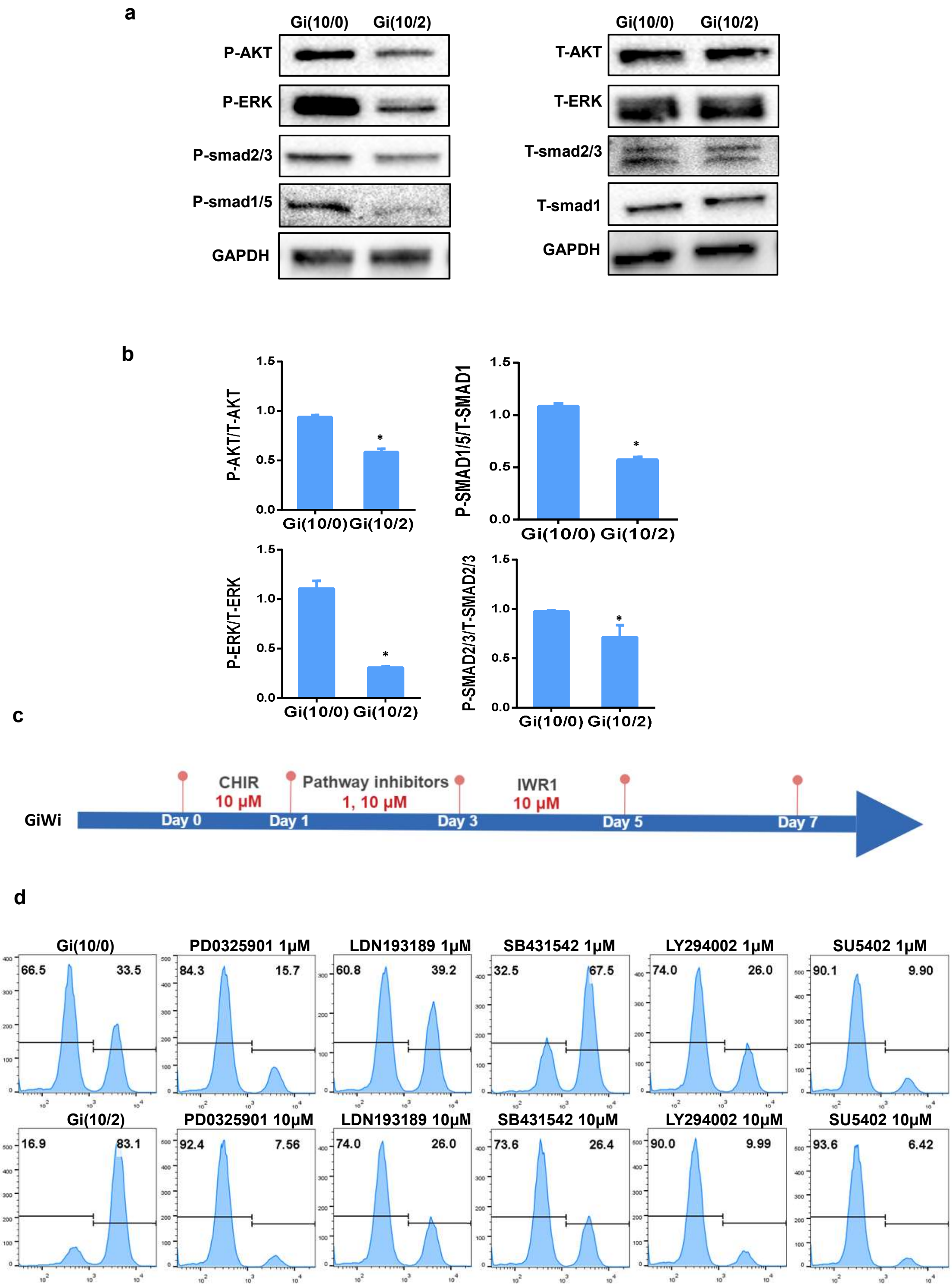

Supplemental Figure 5

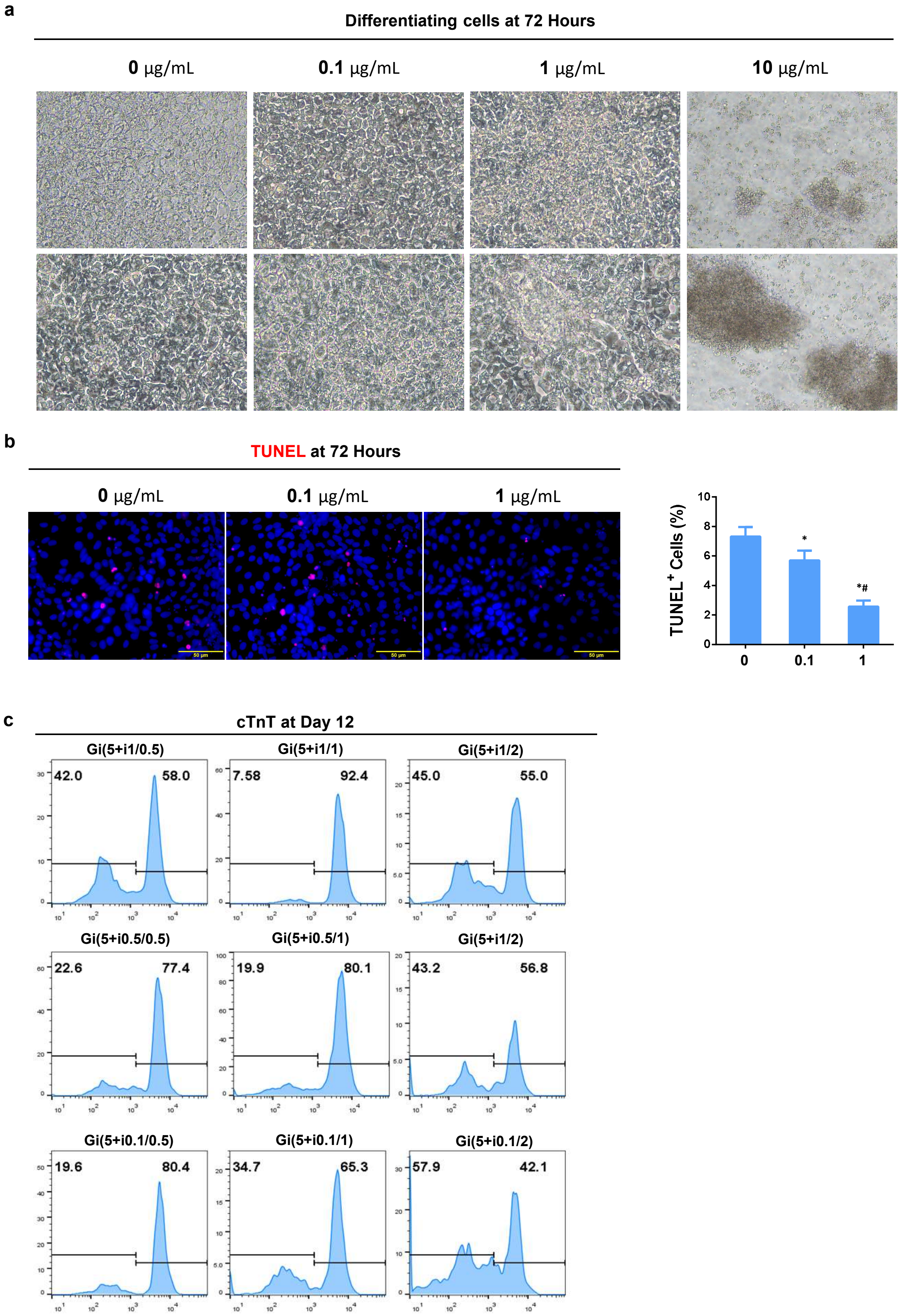

SUPPLEMENTAL FIGURE LEGENDS

Supplemental Figure 1. The conventional GiWi protocol fails to sufficiently promote mesendodermal/mesodermal specification in differentiating hiPSCs. hiPSCs that had been reprogrammed from cardiac fibroblasts (hciPSCs) were differentiated in 10 μM CHIR. **(a-b)** The expression of **(a)** the mesendodermal marker Bry and the Wnt-signaling molecule β-catenin and **(b)** the pluripotency genes OCT4 and SOX2 was evaluated via immunofluorescence before differentiation was initiated and 24 hours afterward; nuclei were counter-stained with DAPI (bars=50 μm). **(c)** Expression of the mesendodermal marker EOMES was evaluated via flow cytometry before differentiation was initiated and 24 hours afterward. **(d)** Expression of the mesodermal marker MESP1 was evaluated via flow cytometry 24 and 72 hours after differentiation was initiated; nuclei were counter-stained with DAPI (bars=100 μm). **(e)** Twelve days after differentiation was initiated, expression of the CM marker cTnT was evaluated in different iPS cell lines, i.e. ciPS, diPS and luc iPS using Gi(10/0) or Gi(10/2) via flow cytometry.

Supplemental Figure 2. The optimized Gi(I/M)Wi protocol limits endodermal and presomitic/paraxial mesodermal commitment in differentiating hiPSCs. **(a)** Expression of the definitive endoderm marker FOXA2 was evaluated in cells from the Gi(10/0), Gi(10/1), Gi(10/2), Gi(10/3), Gi(10/4), and Gi(10/5) groups via immunofluorescence at Hour 72 of differentiation (bar=100 μm); then, the proportion of cells that expressed FOXA2 was calculated and presented as a percentage of the total number of cells (n=3). *$P<0.05$ vs. Gi(10/0). 27 randomly selected fields (3 fields per well) from each group were evaluated. **(b)** Expression of the presomitic mesoderm marker CDX2 and EOMES was evaluated via immunofluorescence in all six treatment groups at Hour 72 of differentiation (bar=50 μm).

Supplemental Figure 3. The optimized Gi(I/M)Wi protocol increases mesendodermal/mesodermal commitment before IWR treatment, and cardiac specification afterward, in differentiating hiPSCs. The expression of **(a)** the mesodermal marker KDR and Bry and **(b)** KDR and the cardiac mesoderm marker PDGFRα was evaluated via flow cytometry in Gi(10/0) and Gi(10/2) cells at the indicated time points after differentiation was initiated.

Supplemental Figure 4. Wnt signaling is the predominant regulator of cardiac mesoderm specification in differentiating hiPSCs. **(a-b)** Protein levels of phosphorylated and total AKT (P-AKT and T-AKT), phosphorylated and total ERK (P-ERK and T-ERK), phosphorylated and total smad2/3 (P-smad2/3 and T-smad2/3), and phosphorylated and total smad1 (P-smad1 and

T-smad1) were **(a)** evaluated via Western blot in Gi(10/0) and Gi(10/2) cells 72 hours after differentiation was initiated and **(b)** presented as the ratio of phosphorylated to total protein (n=3 different batches of differentiated cells). *P<0.05 vs. Gi(10/0). The blots were collected from different gels without cropping. Different blots were separated by white space. **(c)** hiPSCs were differentiated via the GiWi protocol, and 1 or 10 μM concentrations of inhibitors of the PI3K/AKT (LY294002), FGF/ERK (SU5402 and PD0325901), BMP4 (LDN193189), or Smad2/3 (SB431542) pathways were added to the medium from the end of the CHIR treatment phase until the beginning of the IWR1 treatment phase. **(d)** Twelve days after differentiation was initiated, cTnT expression was evaluated via flow cytometry in Gi(10/0) cells and in cells treated with each of the inhibitors. **(e)** Twelve days after differentiation was initiated, expression of the CM marker cTnT was evaluated in luciferase positive cardiac fibroblast derived iPSC clone1 (luc ciPS), wildtype cardiac fibroblast derived iPSC clone 5 (ciPS), and dermal fibroblast derived iPSC (dips) from Gi(10/0) and Gi(10/2) treatment groups via flow cytometry

Supplemental Figure 5. The concentration of insulin treatment affects the cell viability during differentiation. **(a)** Representative bright-field images of differentiating cells with different concentration of insulin treatments 72 hours after differentiation initiation (bar=20 μm). **(b)** Cells treated with different insulin concentrations were TUNEL-stained, and nuclei were counter-stained with DAPI (bar=50 μm); the number of TUNEL$^+$ cells were determined and expressed as a percentage of the total number of cells (n=3 different batches of differentiated cells). 27 randomly selected fields (3 fields per well) from each group were evaluated. *P<0.05 vs 0 μg/mL, #P<0.05 vs 0.1 μg/mL. **(c)** cTnT expression at day 12 was evaluated in cells from 9 different Gi(I+/M)Wi treatment groups via flow cytometry. Initiation concentration for all groups is 5 μM. The insulin treatments are tested at 1μg/mL, 0.5μg/mL and 1 μg/mL with maintenance CHIR concentration at 0.5 μM, 1 μM and 2 μM.

Video S1. Cardiomyocytes differentiated with either no maintenance CHIR treatment (Gi(10/0)).
Video S2. Cardiomyocytes differentiated with low concentration CHIR maintenance (Gi(10/2)).
Video S3. Cardiomyocytes differentiated with insulin.
Video S4. Cardiomyocytes differentiated without insulin.
Video S5. Cardiomyocytes differentiated in suspended culture shot with 10x magnification.
Video S6. Cardiomyocytes differentiated in suspended culture shot with 40x magnification.

SUPPLEMENTAL TABLES

Supplemental Table 1. Antibodies.

Antibody	Source	Identifier
Mouse Monoclonal α-Actinin	Sigma	A7811
Mouse Monoclonal Human cTnT	R&D Systems	MAB1874
Mouse Monoclonal Cardiac Troponin T	Thermo Fisher	RD2196076
Mouse Monoclonal CDX2	Abcam	ab86949
Mouse Monoclonal Phospho-Erk1/2	Cell signaling	9106S
Mouse monoclonal Anti-human Nucleolin	Abcam	ab198580
Mouse Monoclonal MYH6	R&D Systems	MAB8979
Mouse monoclonal EOMES	R&D Systems	IC6166A
Rabbit Monoclonal Phospho-Smad1/5	Cell signaling	9516S
Rabbit Polyclonal Smad1	Cell signaling	6944T
Rabbit polyclonal N-cadherin	Abcam	ab18203
Rabbit polyclonal Connexin 43	Abcam	ab11370
Rabbit polyclonal SOX2	Abcam	ab97959
Rabbit monoclonal OCT4	Abcam	ab209035
Rabbit monoclonal cyclin D1	Abcam	ab134175
Rabbit polyclonal MYL2	Proteintech	10906-1-AP
Rabbit polyclonal Akt	Cell signaling	9272S
Rabbit Monoclonal Phospho-Akt	Cell signaling	4060S
Rabbit Monoclonal Erk1/2	Cell signaling	4695S

Rabbit Monoclonal MYH7	R&D Systems	MAB90961
Rabbit polyclonal CDK4	Santa Cruz	sc-260
Rabbit polyclonal Ki67	EMD Millipore	Ab9260
Rabbit polyclonal phosphor-Histone H3	EMD Millipore	06-570
Rabbit Monoclonal Phospho-Smad2/3	R&D Systems	MAB8935
Rabbit polyclonal Eomes	Abcam	ab23345
Rabbit polyclonal β-catenin	Abcam	ab6302
Rabbit polyclonal Mesp1	Abcam	ab129387
Goat SOX17 NL637-Conjugated	R&D Systems	Cat# SC022
Goat polyclonal HAND1	R&D Systems	AF3168
Goat polyclonal Brachyury	R&D Systems	IC2085A
Goat polyclonal Brachyury	R&D Systems	AF2085
Goat polyclonal Smad2/3	R&D Systems	AF3797
Rat monoclonal VEGF R2/KDR/Flk-1	R&D Systems	FAB4432P
Fitc-donkey anti-mouse	jacksonimmuno	715-095-150
Cy3-donkey anti-rabbit	jacksonimmuno	711-165-152
cy5-donkey anti-mouse	jacksonimmuno	715-175-150

Supplemental Table 2. Reagents, animals and software

**Name**	**Source**	**Identifier**
**Chemicals, Peptides, and Recombinant Proteins**		
Fetal bovine serum (FBS)	Atlanta Biologicals	S11150

Y-27632	Stemcell	72304
Dnase	Fisher scientific	PR-M6101
Donkey Serum	Sigma	D9663
Dimethyl sulfoxide (DMSO)	Sigma	D2438
TRIzol reagent	Fisher scientific	15596018
CHIR99021	Stemcell	72052
IWR1	Stemcell	72562
Protein Extraction Reagent	Fisher scientific	PI78501
Protease and Phosphatase Inhibitor	Fisher scientific	PI78442
insulin	Sigma	91077C
SYBR Green	Fisher scientific	4385617
B27 supplement	Fisher scientific	17-504-044
B27 supplement minus insulin	Fisher scientific	A1895601
LDN193189	Stemcell	1435934-00-1
LY294002	Millipore	154447-36-6
PD0325901	Stemcell	391210-10-9
SU5402	Stemcell	215543-92-3
Bovine serum albumin	Sigma	A3803
Aceton	Fisher scientific	S25904
**Critical Commercial Assays**		
Reverse Transcription Kit	Fisher scientific	4304134
In Situ Cell death detection Kit	Sigma	12156792910

**Experimental Models: Cell Lines**		
Human cardiac fibroblast-derived iPS	This paper	N/A
Human dermal fibroblast-derived iPS	This paper	N/A
**Experimental Models: Organisms/Strains**		
NOD/SCID Mice	Jackson Lab	001303
**Software and Algorithms**		
FlowJo	Flowjo, LLC	N/A
Graphpad Prism v.6.01	Graphpad	N/A
Image J	NIH	https://imagej.nih.gov/ij/

Supplemental Table 3. Primers

Primer	Application	Sequence
Bry-FW	qRT-PCR	5'-TGCTTCCCTGAGACCCAGTT-3'
Bry-RV	qRT-PCR	5'-GATCACTTCTTTCCTTTGCATCAAG-3'
KDR-FW	qRT-PCR	5'-CCCCAGAAATAAAATGGTATAAAAATG-3'
KDR-RV	qRT-PCR	5'-TTTCACTCACTTCCATAATCGTCA-3'
ISL1-FW	qRT-PCR	5'-AGATTATATCAGGTTGTACGGGATCA-3'
ISL1-RV	qRT-PCR	5'-ACACAGCGGAAACACTCGAT-3'
MESP1-FW	qRT-PCR	5'-GAAGTGGTTCCTTGGCAGAC-3'
MESP1-RV	qRT-PCR	5'-TCCTGCTTGCCTCAAAGTGT-3'

NKX2.5-FW	qRT-PCR	5'-CAAGTGTGCGTCTGCCTTT-3'
NKX2.5-RV	qRT-PCR	5'-CAGCTCTTTCTTTTCGGCTCTA-3'
PAX1-FW	qRT-PCR	5'-TCGCTATGGAGCAGACGTATG-3'
PAX1-RV	qRT-PCR	5'-GCTGCCGACTGATGTCACA-3'
SOX17-FW	qRT-PCR	5'-AGATGCTGGGCAAGTCGT-3'
SOX17-RV	qRT-PCR	5'-GCTTCAGCCGCTTCACC-3'
SOX2-FW	qRT-PCR	5'-TGGACAGTTACGCGCACAT-3'
SOX2-RV	qRT-PCR	5'-CGAGTAGGACATGCTGTAGGT-3'
TCF15-FW	qRT-PCR	5'-GCACCTTCTGCCTCAGCAACCAGC-3'
TCF15-RV	qRT-PCR	5'-GGTCCCCCGGTCCCTACACAA-3'
CDX1-FW	qRT-PCR	5'-GGTGGCAGCGGTAAGACTC-3'
CDX1-RV	qRT-PCR	5'-TGTAACGGCTGTAATGAAACTCC-3'
CDX2-FW	qRT-PCR	5'-GGAACCTGTGCGAGTGGAT-3'
CDX2-RV	qRT-PCR	5'-TCGATATTTGTCTTTCGTCCTG-3'
EOMES-FW	qRT-PCR	5'-CACATTGTAGTGGGCAGTGG-3'
EOMES-RV	qRT-PCR	5'-CGCCACCAAACTGAGATGAT-3'
FOXA2-FW	qRT-PCR	5'-ATTGCTGGTCGTTTGTTGTG-3'
FOXA2-RV	qRT-PCR	5'-CCTCGGGCTCTGCATAGTAG-3'
HAND1-FW	qRT-PCR	5'-GTGAGAGCAAGCGGAAAAG-3'
HAND1-RV	qRT-PCR	5'-GTGCGTCCTTTAATCCTCTTC-3'
GATA4-FW	qRT-PCR	5'-TGCCGTTCATCTTGTGGTAG-3'
GATA4-RV	qRT-PCR	5'-CCGACACCCCAATCTCG-3'

OCT4-FW	qRT-PCR	5'-CAGTGCCCGAAACCCACAC-3'
OCT4-RV	qRT-PCR	5'-GGAGACCCAGCAGCCTCAAA-3'
